# A nomogram for predicting adverse pathologic features in low-risk papillary thyroid microcarcinoma

**DOI:** 10.1186/s12885-024-12012-3

**Published:** 2024-02-22

**Authors:** Lei Gong, Ping Li, Jingjing Liu, Yan Liu, Xinghong Guo, Weili Liang, Bin Lv, Peng Su, Kai Liang

**Affiliations:** 1 Department of Endocrinology and Metabolic Diseases, Qilu Hospital of Shandong University, Jinan, China; 2 Department of Endocrinology, Ningyang Second People’s Hospital, Jinning, China; 3 Department of Thyroid Surgery, General Surgery, Qilu Hospital of Shandong University, Jinan, China; 4 Department of Pathology, Qilu Hospital of Shandong University, Jinan, China

**Keywords:** Low-risk papillary thyroid microcarcinoma, Nomogram, Adverse pathologic features

## Abstract

**Background:**

Identifying risk factors for adverse pathologic features in low-risk papillary thyroid microcarcinoma (PTMC) can provide valuable insights into the necessity of surgical or non-surgical treatment. This study aims to develop a nomogram for predicting the probability of adverse pathologic features in low-risk PTMC patients.

**Methods:**

A total of 662 patients with low-risk PTMC who underwent thyroid surgery were retrospectively analyzed in Qilu Hospital of Shandong University from May 2019 to December 2021. Logistic regression analysis was used to determine the risk factors for adverse pathologic features, and a nomogram was constructed based on these factors.

**Results:**

Most PTMC patients with these adverse pathologic features had tumor diameters greater than 0.6 cm (*p* < 0.05). Other factors (age, gender, family history of thyroid cancer, history of autoimmune thyroiditis, and BRAF^*V600E*^ mutation) had no significant correlation with adverse pathologic features (*p* > 0.05 each). The nomogram was drawn to provide a quantitative and convenient tool for predicting the risk of adverse pathologic features based on age, gender, family history of thyroid cancer, autoimmune thyroiditis, tumor size, and BRAF^*V600E*^ mutation in low-risk PTMC patients. The areas under curves (AUC) were 0.645 (95% CI 0.580–0.702). Additionally, decision curve analysis (DCA) and calibration curves were used to evaluate the clinical benefits of this nomogram, presenting a high net benefit.

**Conclusion:**

Tumor size > 0.60 cm was identified as an independent risk factor for adverse pathologic features in low-risk PTMC patients. The nomogram had a high predictive value and consistency based on these factors.

## Introduction

Thyroid cancer incidence is high in many countries, including low- and middle-income countries, while the mortality rate is low in some countries [1]. The primary reason is the increased incidence of papillary thyroid microcarcinoma (PTMC) [2]. A low-risk PTMC is defined as a papillary thyroid carcinoma (PTC) ≤ 10 mm without significant extrathyroidal extension, lymph node metastasis, or distant metastasis (T1aN0M0).

There are still large differences in the treatment strategies for low-risk PTMC, with surgery, active surveillance, and ultrasound-guided thermal ablation being the primary treatment methods [3–5]. Surgery is the preferred treatment for PTMC. However, it is more traumatic, can easily cause recurrent laryngeal nerve and parathyroid gland injury, impairs thyroid function, and results in obvious postoperative complications [6, 7]. Based on this, Akira Miyauchi's team at Kumma Hospital first proposed active surveillance as an alternative to immediate surgical treatment for low-risk PTMC in 1993 [8]. Active surveillance has been adopted as a recommendation in guidelines published in the United States [3] and Japan [9]. However, active surveillance is not widespread in low-risk PTMC patients. Surgeons and patients acknowledge patient fear and anxiety are reasons to choose surgery instead of active surveillance [10]. Ultrasound-guided thermal ablation is a new surgical method, including radiofrequency ablation, laser ablation, and microwave ablation. Radiofrequency ablation has the advantages of short treatment time, less trauma, and high aesthetics [11, 12]. A multicenter prospective cohort study including 1177 patients with low-risk PTMC (immediate operation versus delayed operation after active surveillance) was conducted at 3 tertiary hospitals in Korea from June 2016 to January 2020. The result suggest that active surveillance might be considered an alternative treatment option for patients with low-risk PTMC regarding the extent of thyroidectomy and postoperative complications [13]. From January 2017 through June 2021, low-risk PTMC patients were screened in a prospective multicenter study. The management details of active surveillance, surgery, and thermal ablation were discussed. Thermal ablation of low-risk PTMC was observed to be safe and efficacious with few minor complications. This technique may help to bridge the gap between surgery and active surveillance as a treatment option [14].

Little is known about which patients are most suitable for non-surgical treatment (active surveillance and radiofrequency ablation). Some PTMC patients exhibit more aggressive features, such as early metastasis and lymph node involvement, affecting their survival. Therefore, a subset of PTMC patients still requires more detailed risk stratification and individualized treatment strategies [15].

We selected PTMC patients who underwent thyroid surgery at Qilu Hospital of Shandong University from May 2019 to December 2021. According to preoperative assessment and postoperative pathological comparison, we performed an adverse pathologic features-predicting nomogram including clinical features of low-risk PTMC patients to provide the basis for formulating individualized treatment strategies.

## Materials and methods

### Study population

**Fig. 1:**
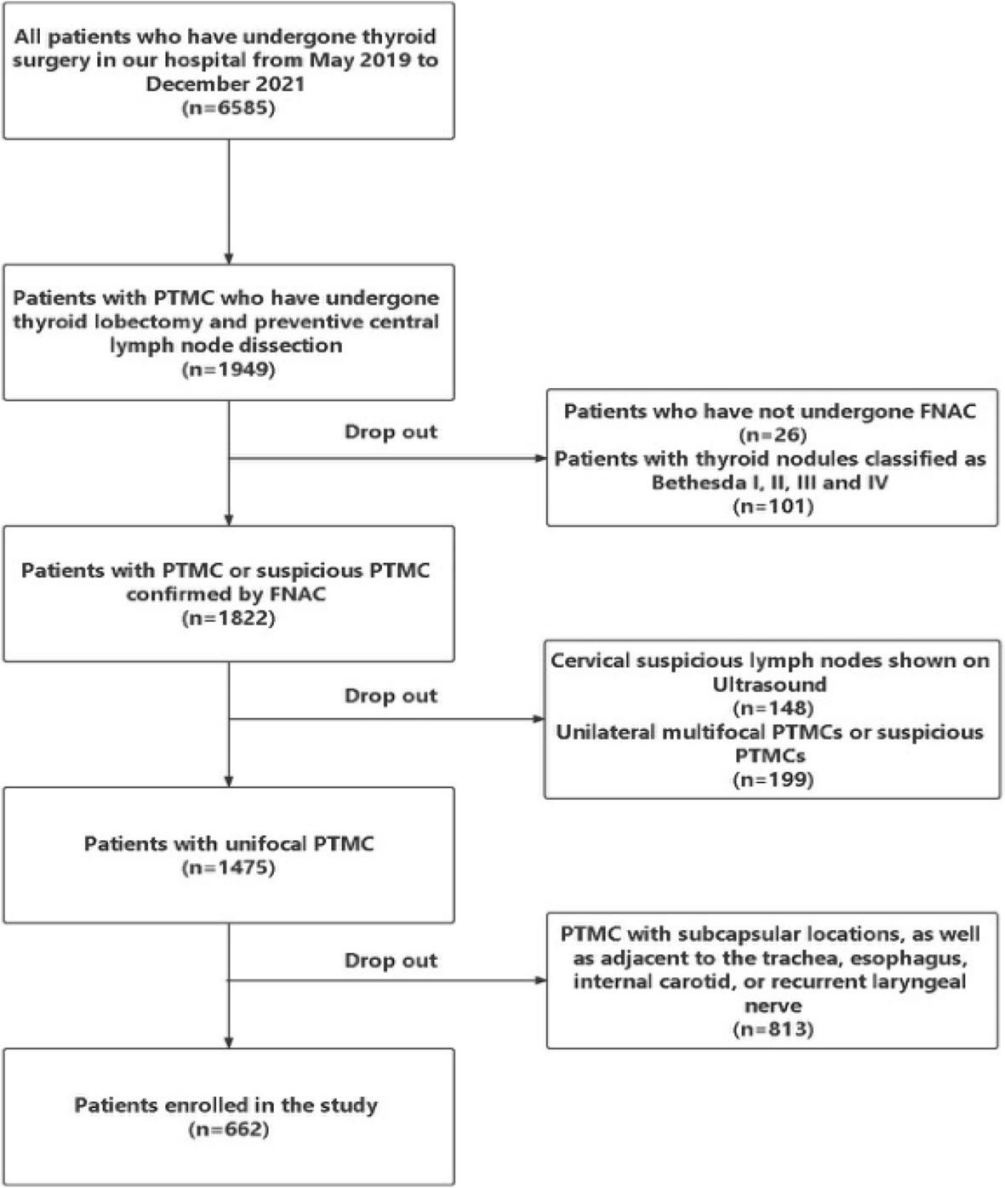
The patient screening flowchart for the present study

This retrospective study was approved by the Ethics Committee of Qilu Hospital of Shandong University (ethical approval number KYLL-2018(KS)-226). A total of 6,585 patients had undergone thyroid surgery in our hospital from May 2019 to December 2021, of whom 5,923 were excluded according to the selection flowchart shown in Fig. 1, leaving a final sample of 662 patients (163 men and 499 women) who had undergone thyroid lobectomy and preventive central lymph node dissection for analysis. The present study’s inclusion criteria were as follows: (i) unifocal PTMC or suspicious PTMC confirmed by fine needle aspiration (FNA); (ii) surrounded by ≥ 2 mm of normal thyroid parenchyma, as well as not adjacent to the trachea, esophagus, internal carotid, or recurrent laryngeal nerve; (iii) no cervical lymph node metastasis (LNM) exhibited on Ultrasound; (iv) no distant metastasis presented on images; and (v) complete clinical data.

### Data source

The following information from His system of Qilu Hospital of Shandong University was collected to establish a retrospective database. Basic information included age, gender, family history of thyroid cancer, history of autoimmune thyroiditis, tumor size, and BRAF^*V600E*^ mutation. Adverse pathologic features included thyroid capsule invasion, extrathyroidal invasion, intraglandular dissemination, aggressive variants, occult thyroid carcinoma, central lymph node metastasis, and several central lymph node metastases.

Cytological classification was based on The Bethesda System for Reporting Thyroid Cytopathology [16]. Real-time polymerase chain reaction (RT-PCR) was used for BRAF^*V600E*^ genetic testing by the BRAF p.V600E Mutations Detection Kit (Amoy Diagnostics Co., Ltd., Xiamen, China). Post-surgical pathological diagnosis was based on the WHO Classification of Tumours of Endocrine Organs (2017).

### Statistical analysis

Continuous variables were expressed as mean ± SD and compared using an unpaired T-test or Kruskal–Wallis test. The categorical variables were expressed numerically (proportioned). The χ^2^ test was used to compare groups. Multivariate logistic regression analysis was used to determine the independent risk factors for adverse pathologic features. Based on multivariate logistic regression analysis results, a nomogram was established to predict adverse pathologic features. The areas under curves (AUC) of receiver operating characteristic (ROC) constructed by bootstrap resampling (times = 500) were used to evaluate the prediction of adverse pathologic features for internal verification of this nomogram. Calibration curves and decision curve analysis (DCA) curves were used to verify the calibration and clinical practicability of the nomogram, respectively. Statistical analysis was bilateral, and differences with *p*-values < 0.05 were considered statistically significant. All statistical analyses were established using R version 3.6.3 (http://www.R-project.org) and EmpowerStats software (www.empowerstats.com, X&Y solutions, Inc. Boston, MA, USA).

## Results

### Clinical characteristics of the study population

This study included 662 participants. Participants were divided into two groups: one without adverse pathologic features (*n* = 500) and the other with adverse pathologic features (*n* = 162). Only tumor size (0.58 ± 0.19 cm vs. 0.64 ± 0.18 cm, *p* < 0.001) was statistically significant between the two groups, while age (44.14 ± 11.31 years old vs. 42.35 ± 11.56 years old, *p* = 0.082), gender (*p* = 0.056), family history of thyroid cancer (*p* = 0.105), history of autoimmune thyroiditis (*p* = 0.816), and BRAF^*V600E*^ mutation (*p* = 0.81) were not. Table 1 summarizes the specific demographic and clinical characteristics of low-risk PTMC patients.

**Table 1:** Characteristics of the study population who presented with low risk papillary thyroid microcarcinoma

		**Adverse pathologic features**		
**Characteristic**	**Study population**	**Not present**	**Present**	***P* value**
	**(*n* = 662)**	**(*n* = 500)**	**(*n* = 162)**	
Age, years	43.70 ± 11.39	44.14 ± 11.31	42.35 ± 11.56	0.082
Age categories				0.515
< 40	268 (40.48%)	197 (39.40%)	71 (43.83%)	
40–59	339 (51.25%)	259 (51.80%)	80 (49.38%)	
≧60	55 (8.31%)	44 (8.80%)	11 (6.79%)	
Gender				0.056
Male	163 (24.62%)	114 (22.80%)	49 (30.25%)	
Female	499 (75.38%)	386 (77.20%)	113 (69.75%)	
Family history of thyroid cancer				0.105
Yes	17 (2.57%)	10 (2.00%)	7 (4.32%)	
No	645 (97.43%)	490 (98.00%)	155 (95.68%)	
Autoimmune thyroiditis				0.816
Yes	135 (20.39%)	103 (20.60%)	32 (19.75%)	
No	427 (79.61%)	397 (79.40%)	130 (80.25%)	
Tumor size, cm	0.59 ± 0.19	0.58 ± 0.19	0.64 ± 0.18	< 0.001
Tumor size categories				0.016
≤ 0.40	149 (22.51%)	124 (24.80%)	25 (15.43%)	
0.41–0.60	248 (37.46%)	190 (38.00%)	58 (35.80%)	
0.61–0.80	174 (26.28%)	126 (25.20%)	48 (29.63%)	
0.81–1.00	91 (13.75%)	60 (12.00%)	31 (19.14%)	
BRAF^*V600E*^ mutation				0.81
Positive	369 (55.74%)	274 (54.80%)	95 (58.64%)	
Negative	40 (6.04%)	29 (5.8%)	11 (6.79%)	
N/A	253 (38.22%)	197 (39.40%)	56 (34.57%)	

### Risk of adverse pathologic features in the study population

The risk of adverse pathological features in the study population (*n* = 662) mainly included the following: (i) thyroid capsule invasion was present in 32 cases (4.83%); (ii) intraglandular dissemination was present in 17 cases (2.57%); (iii) aggressive variants were present in 27 cases (4.08%), most of which were tall cell variant (3.78%); (iv) occult thyroid carcinoma was present in 35 cases (5.29%); and (v) central lymph node metastasis was present in 83 cases (12.54%), in most of them, the number of lymph node metastases was one. No patients had an extrathyroidal invasion. Table 2 illustrates the data.

**Table 2:** Risk of adverse pathologic features in patients with low risk papillary thyroid microcarcinoma

Variable	Study population
	**(*n* = 662)**
Thyroid capsule invasion	32 (4.83%)
Extrathyroidal invasion	0
Intraglandular dissemination	17 (2.57%)
Aggressive variants	27 (4.08%)
Tall cell variant	25 (3.78%)
Diffuse sclerosing variant	1 (0.15%)
Hobnail variant	1 (0.15%)
Occult thyroid carcinoma	35 (5.29%)
Central lymph node metastasis	83 (12.54%)
Number of central lymph node metastasis	
1	50 (7.55%)
2	19 (2.87%)
≥ 3	14 (2.11%)

### Characteristics of the study population with adverse pathologic features

**Fig. 2:**
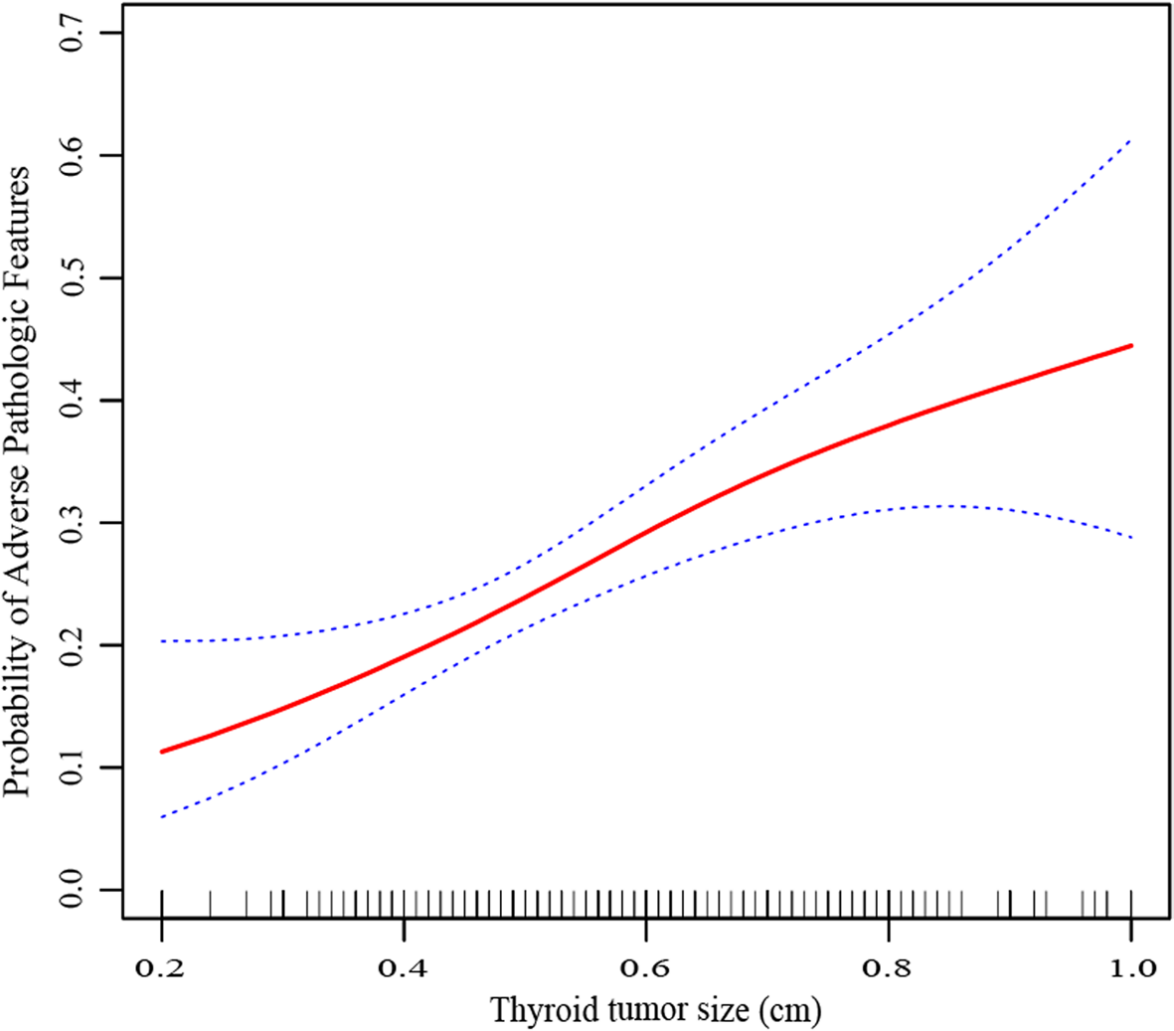
The general additive model demonstrates the relationship between thyroid tumor sizes and the risk of adverse pathologic features in low-risk PTMC patients. The resulting figure displays the predicted adjusted probability on the y-axis and thyroid tumor size on the x-axis

Most PTMC patients with these adverse pathologic features had tumor diameters greater than 0.6 cm (*p* < 0.05). Other factors (age, gender, family history of thyroid cancer, history of autoimmune thyroiditis, BRAF^*V600E*^ mutation) had no significant correlation with adverse pathologic features (*p* > 0.05 each). The results were consistent after multivariate correction analysis (Table 3). The general additive model demonstrates the relationship between thyroid tumor sizes and the risk of adverse pathologic features in low-risk PTMC patients (Fig. 2). The larger the tumor diameter, the higher the probability of adverse pathologic features.

**Table 3:** Characteristics of low-risk PTMC patients with adverse pathologic features

Characteristic	Probability of adverse pathologic features	No-adjusted odds ratio	*p* Value	Adjusted odds ratio	*p* Value
		**(95% CI)**		**(95% CI)**	
Age categories					
< 40	26.49%	1.44 (0.71, 2.94)	0.316	1.14 (0.44, 2.96)	0.787
40–59	23.60%	1.24 (0.61, 2.50)	0.558	1.22 (0.48, 3.11)	0.677
≧60	20.00%	Reference	-	Reference	-
Gender					
Male	30.06%	1.47 (0.99, 2.18)	0.057	1.28 (0.75, 2.17)	0.366
Female	22.64%	Reference	-	Reference	-
Family history of thyroid cancer					
0	24.03%	Reference	-	Reference	-
1	41.18%	2.21 (0.83, 5.91)	0.113	2.14 (0.64, 7.19)	0.217
Autoimmune thyroiditis					
0	30.44%	Reference	-	Reference	-
1	23.70%	0.95 (0.61, 1.48)	0.816	1.36 (0.77, 2.41)	0.286
Tumor size categories					
≤ 0.40	16.78%	Reference	-	Reference	-
0.41–0.60	23.39%	1.51 (0.90, 2.55)	0.118	1.58 (0.85, 2.94)	0.147
0.61–0.80	27.59%	1.89 (1.10, 3.25)	0.022	2.85 (1.48, 5.51)	0.002
0.81–1.00	34.07%	2.56 (1.39, 4.72)	0.003	3.24 (1.46, 7.16)	0.004
BRAF^*V600E*^ mutation					
Negative	27.50%	Reference	-	Reference	-
Positive	25.75%	0.91 (0.44, 1.90)	0.809	1.05 (0.49, 2.25)	0.895

### Development and validation of an adverse pathologic features-predicting nomogram

**Fig. 3:**
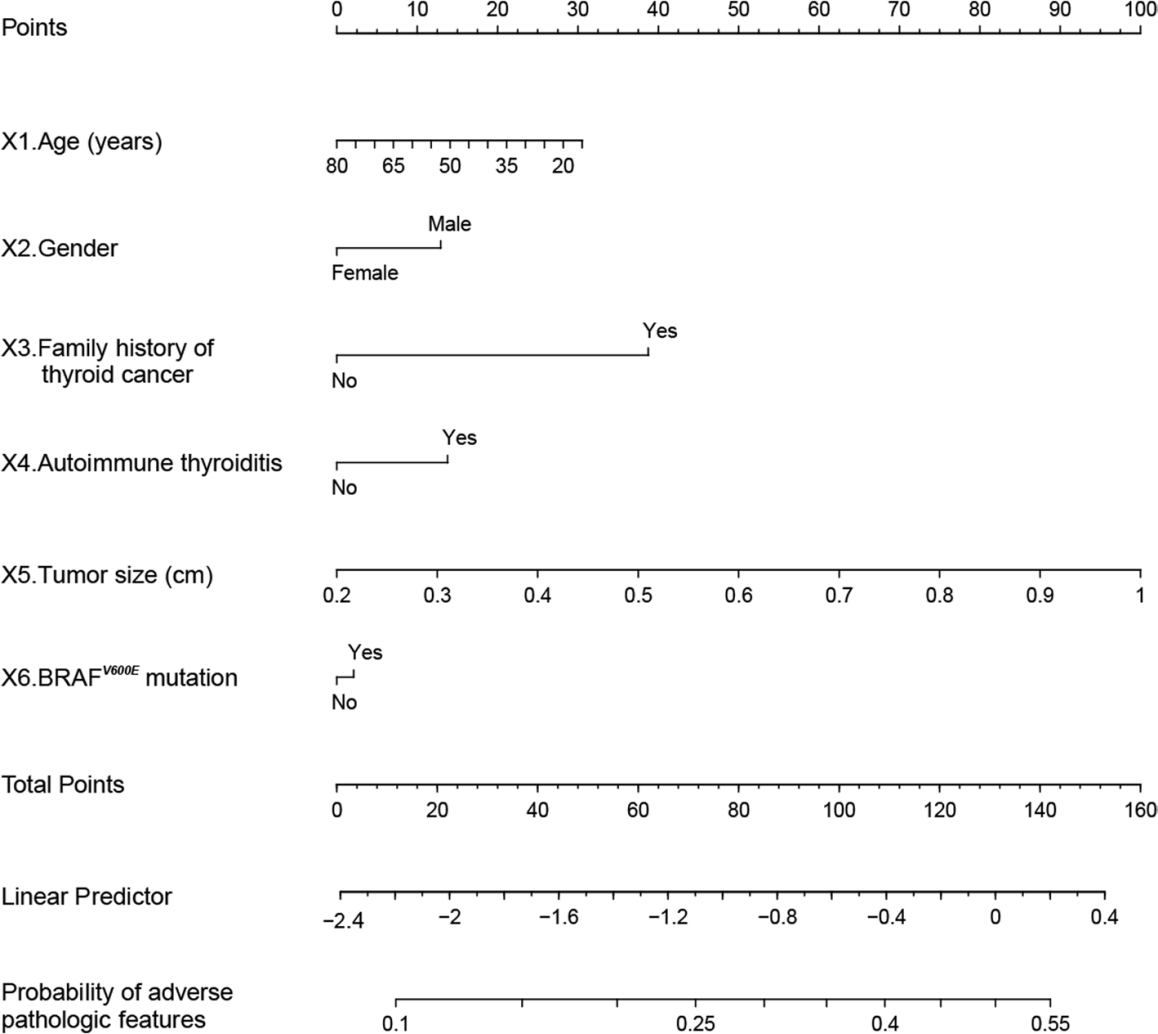
Nomogram used for preoperatively predicting the adverse pathologic features in low-risk PTMC patients. The nomogram consists of graph lines that include six risk factors (Age, Gender, Family history of thyroid cancer, Autoimmune thyroiditis, Tumor size, and BRAFV600E mutation), individual scores (Points), total scores (Total Points), and event risk (Adverse pathologic features). The line segment corresponding to each risk factor is marked with a scale, which represents the range of possible values of the factor, and the length of the line segment reflects the factor's contribution to the outcome event. “Points” at the top of the graph indicate the corresponding scores of risk factors under different values. The total score of all the individual scores of the risk factors is “Total Points,” which corresponds to “Adverse pathologic features” at the bottom of the graph, representing the predicted probability of adverse pathologic features

The nomogram was drawn to provide a quantitative and convenient tool for predicting the risk of adverse pathologic features in low-risk PTMC patients based on age, gender, family history of thyroid cancer, autoimmune thyroiditis, tumor size, and BRAF^*V600E*^ mutation (Fig. 3). Predictive Model: Logit (Adverse pathologic features) = -1.70707—0.00864*Age—0.23753*Gender + 0.71291*Family history of thyroid cancer + 0.25380*Autoimmune thyroiditis + 2.30019*Tumor size + 0.03822*BRAF^*V600E*^ mutation. Table 4 presents the prediction accuracy of the nomogram. The nomogram score/predicted probability was a numeric value representing the prediction model score of the patient. For example, sensitivity was 10.56%, specificity was 99.05%, positive predictive value was 27.02%, and negative predictive value was 96.96% using a cutoff value 0.15.

**Table 4:** Predictive value of nomogram scores at different cutoff points

Nomogram Score/Predicted Probability	Threshold/Linear Predictor	Sensitivity (%)	Specificity (%)	Positive Predictive Value (%)	Negative Predictive Value (%)
≥ 0.15	-1.74	10.56	99.05	27.02	96.96
≥ 0.20	-1.38	35.97	83.01	31.20	85.82
≥ 0.25	-1.10	60.39	60.37	34.78	81.33
≥ 0.30	-0.84	73.59	38.67	33.88	77.43
≥ 0.35	-0.61	83.82	23.58	33.78	75.82
≥ 0.40	-0.40	92.73	17.92	46.34	76.35

**Fig. 4:**
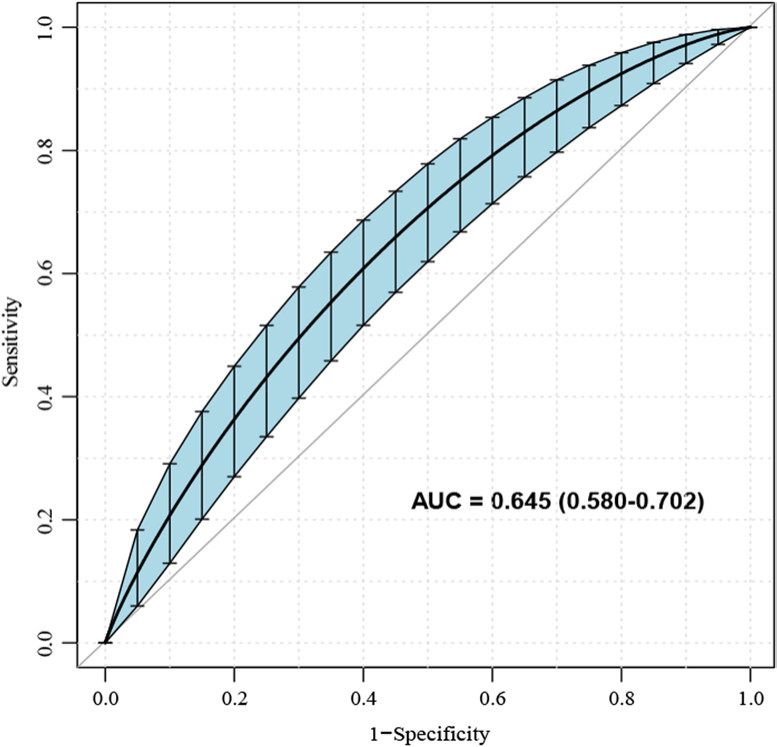
The ROC curve after internal validation using bootstrap resampling (times = 500). The area under the ROC curve (AUC) was 0.645 (95% CI 0.580–0.702). Blue shading shows the bootstrap estimated 95% CI with the AUC

**Fig. 5:**
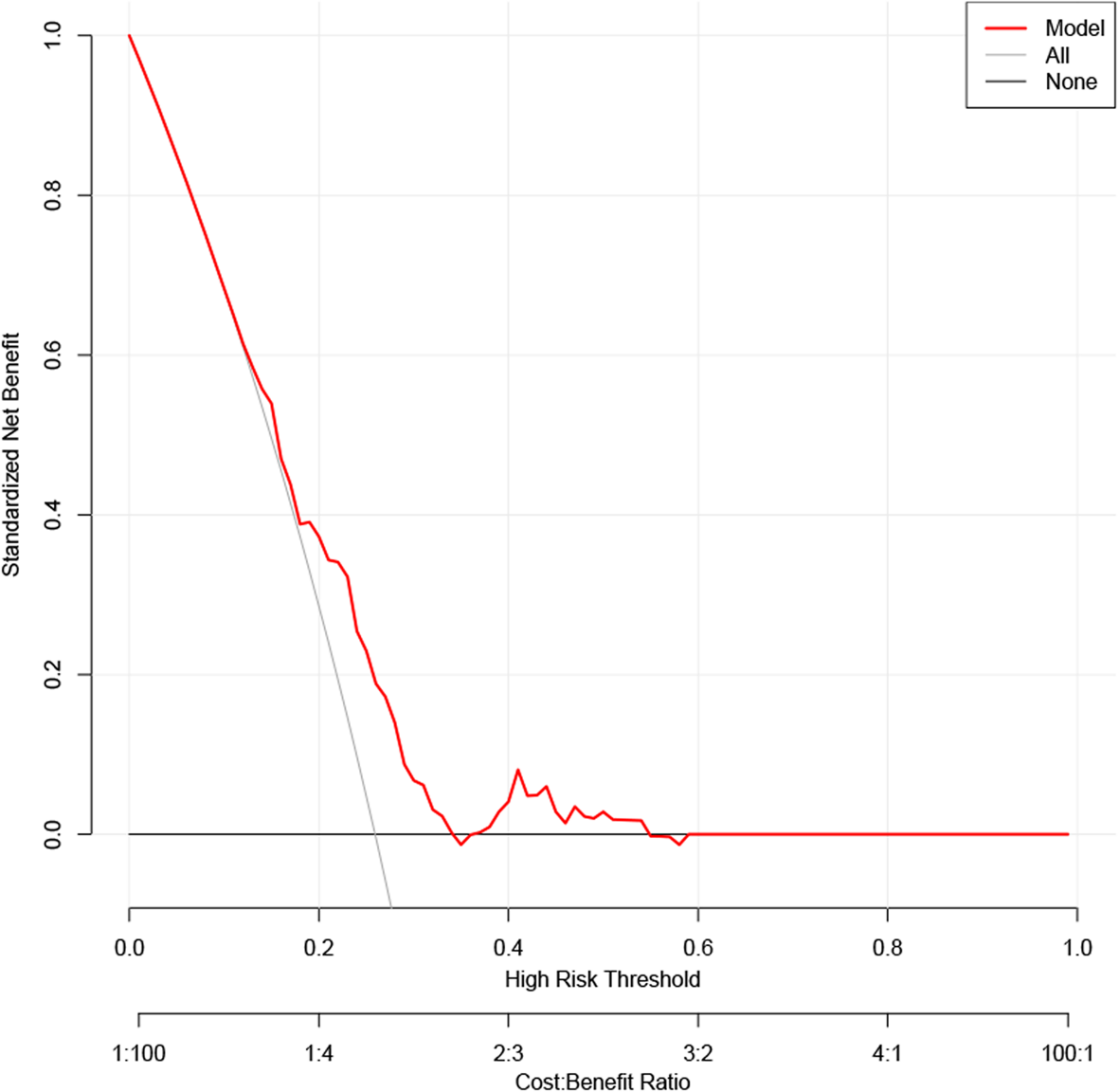
The DCA of the model for preoperatively predicting the adverse pathologic features. Net benefit curve of the predictive model. “None” line = net benefit when no participant is considered as having the outcome (adverse pathologic features); “All” line = net benefit when all participants are considered as having the outcome. The preferred model has the highest net benefit at any threshold

**Fig. 6:**
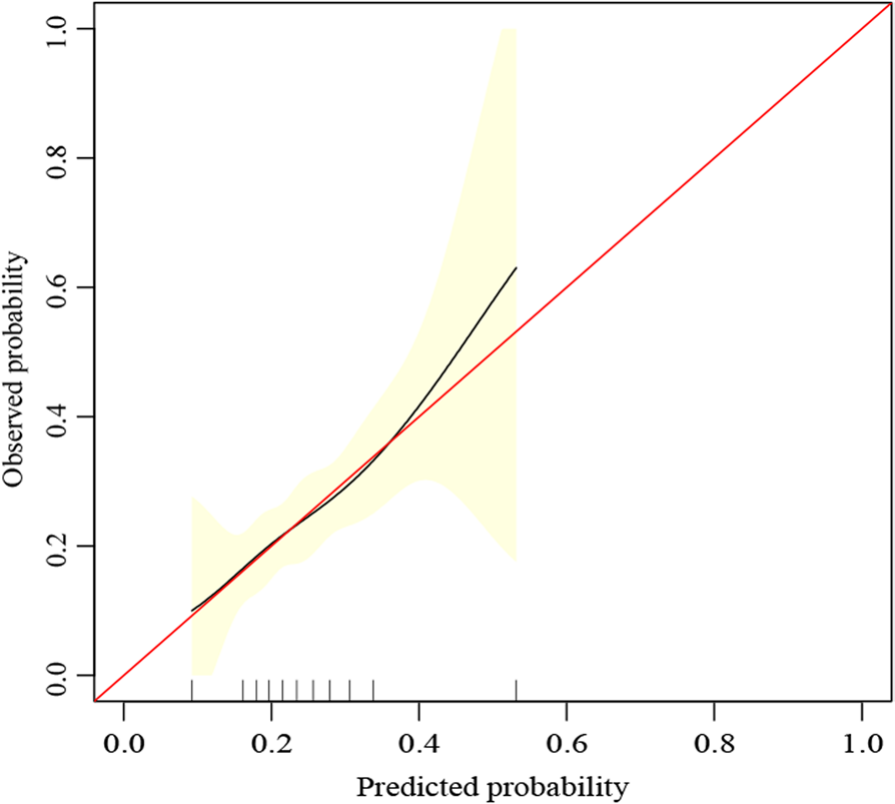
Calibration curve for preoperatively predicting the probability of adverse pathologic features

The AUC of ROC constructed by bootstrap resampling (times = 500) was used to evaluate the prediction of adverse pathologic features for internal verification of this nomogram. The AUC was 0.645 (95% CI 0.580–0.702) (Fig. 4). Additionally, DCA (Fig. 5) and calibration curves (Fig. 6) were used to evaluate the clinical benefits of this nomogram. This nomogram exhibited a high net benefit, especially for predicted probability thresholds between 0 and 35%.

Predictive Model: Logit (Adverse pathologic features) = -1.70707—0.00864*Age—0.23753*Gender + 0.71291*Family history of thyroid cancer + 0.25380*Autoimmune thyroiditis + 2.30019*Tumor size + 0.03822*BRAFV600E mutation.

## Discussion

Thyroid cancer incidence has increased substantially over the last few decades, primarily driven by increases in papillary thyroid cancer [17]. The increased incidence may be due to the widespread use of highly sensitive diagnostic tools, such as ultrasound, and the popularity of health examinations, which can detect smaller nodules, especially PTMC. The latest statistics from the United States present that 10-year disease-specific survival (DSS) for differentiated thyroid cancer (DTC) increased over time (95.4% for patients diagnosed in 1992–1998, 96.6% in 1999–2008, and 97.3% in 2009–2018), while 10-year DSS for DTC with distant metastases remained unchanged (50.2%, 47.3%, and 52.4%, respectively) [18]. The over-diagnosis of PTMC has led to an emerging trend of narrow surgery and an inclination toward active surveillance when possible [3]. However, the 2015 American Thyroid Association guidelines present that a subset of PTMC has local and distant metastases, and there are currently no clinically reliable criteria to identify these patients [3]. Some of these features, such as lymphovascular invasion and microscopic extrathyroidal extension, are frequently undetectable by preoperative ultrasound and cytology alone, while others may be missed by routine tests [15]. The presence of lymph node metastasis and the extent of extranodal invasion are always associated with an unfavorable tumor prognosis [19, 20]. Therefore, it is necessary to be aware of the risks associated with adverse pathologic features of PTMC.

This study selected patients with low-risk PTMC who met the requirements in Fig. 1, and non-surgical treatment, including active surveillance and radiofrequency ablation, was suitable for these nodules according to the guidelines [3, 4, 9, 21]. According to the postoperative pathological results of these patients, the relevant factors of adverse pathologic features were analyzed retrospectively, and an adverse pathologic features-predicting nomogram was developed to screen the patients suitable for non-surgical treatment. It provides a reference for clinicians to choose the appropriate treatment plan. We focus on predicting central lymph node metastasis (CLNM) and lateral lymph node metastasis (LLNM) in PTMC to provide evidence for lymph node dissection, differing significantly from other published studies [22–24]. Our nomogram provides a basis for selecting low-risk PTMC patients more suitable for non-surgical treatment. The accurate selection of patients for non-surgical treatment requires rigorous screening of low-risk PTMC, but it must be recognized that there are no precise screening criteria. Tuttle et al. proposed a clinical framework for PTMC active surveillance by evaluating the important characteristics of three interrelated domains (pre-operative imaging/clinical findings, patient characteristics, and medical team characteristics) to classify patients as ideal, appropriate, or inappropriate for active surveillance [25]. We developed inclusion criteria by referring to the population criteria for ideal active surveillance in this clinical framework.

Our results display that most PTMC patients with these adverse pathologic features had tumor diameters greater than 0.6 cm (*p* < 0.05). Other factors (age, gender, family history of thyroid cancer, history of autoimmune thyroiditis, and BRAF^*V600E*^ mutation) had no significant correlation with adverse pathologic features (*p* > 0.05 each). The larger the tumor diameter, the higher the probability of adverse pathologic features. This is consistent with the results reported in other literatures [22, 24, 26]. Tumor size is a prognostic factor for differentiated thyroid carcinoma [3]. Previous studies have reported a significant correlation between increased cervical lymph node metastasis incidence and tumor size. For PTMC patients, the tangential values of meaningful tumor size were not uniform. A previous study reported that tumor size > 6 mm was an independent predictor of the high prevalence of CLNM [26]. Liu et al. retrospectively analyzed 4,872 patients with cN0 unifocal PTMC and discovered that greater than 7 mm in size was an independent risk factor for LLNM [24]. Our study divided patients into four groups according to tumor size: ≤ 0.40 cm, 0.41–0.60 cm, 0.61–0.80 cm, and 0.81–1.00 cm. Statistical analysis determined that the meaningful cutoff value of tumor size was 0.60 cm. The probability of adverse pathologic features was 16.78%, 23.39%, 27.59%, and 34.07% in the four groups, respectively. Tumor size > 0.60 cm was identified as an independent risk factor for adverse pathologic features. Tumor size cutoff values may differ across studies due to differences in inclusion criteria and sample sizes.

Previous studies on PTMC have demonstrated that factors related to adverse pathologic features mainly include age, gender, family history of thyroid cancer, autoimmune thyroiditis, and BRAF^*V600E*^ mutation [3, 27–31]. This differs from our study’s results, possibly due to our relatively small sample size, inclusion criteria, and differences in a research environment. Therefore, these results must be further evaluated in multi-center studies with larger sample sizes. However, based on the previous studies, we developed a nomogram for preoperatively predicting the adverse pathologic features in low-risk PTMC patients. The nomogram consists of graph lines that include six risk factors (Age, Gender, Family history of thyroid cancer, Autoimmune thyroiditis, Tumor size, and BRAF^*V600E*^ mutation), individual scores (Points), total scores (Total Points), and event risk (Adverse pathologic features). Predictive Model: Logit (Adverse pathologic features) = -1.70707—0.00864*Age—0.23753*Gender + 0.71291*Family history of thyroid cancer + 0.25380*Autoimmune thyroiditis + 2.30019*Tumor size + 0.03822*BRAF^*V600E*^ mutation. The AUC of ROC constructed by bootstrap resampling (times = 500) was used to evaluate the prediction of adverse pathologic features for internal verification of this nomogram. The AUC was 0.645 (95% CI 0.580–0.702). Additionally, DCA (Fig. 5) and calibration curves (Fig. 6) were used to evaluate the clinical benefits of this nomogram. The results display that our nomogram has a good predictive effect. DCA and calibration curves also present clinical practicability and satisfactory accuracy.

Incorporating clinical features into an easy-to-use nomogram enables individualized prediction of adverse pathologic features before surgery. In a study of osteosarcoma, authors visualized the pseudogene signature and the other clinical information by a nomogram to simplify the use of this signature in clinical practice [32]. This study’s nomogram may help to determine the presence of adverse pathologic features and avoid over-treatment and under-treatment. Based on our findings, we recommend surgical treatment for PTMC patients at high risk for adverse pathologic features. Simultaneously, patients with a low risk of adverse pathologic features should receive non-surgical treatment to avoid possible surgical complications.

This study still has some limitations. First, the disadvantage of our nomogram is the lack of external validation, limiting its clinical application in other regions. Additional external validation cohorts of prospective studies are urgently needed to evaluate the feasibility of our nomogram further. Second, avoiding selection bias is difficult because this is a single-center retrospective study. This study has the limitations of small number of patients enrolled and short time span of data collection. At the same time, racial differences, regional differences, and differences in treatment concepts may have an impact on the results. A multicenter study is planned in the future to reduce potential bias.Third, our nomogram only includes six variables, suggesting that potential variables may be discovered to make our nomogram more complete and reliable in future practice.

## Conclusion

In conclusion, tumor size > 0.60 cm was identified as an independent risk factor for adverse pathologic features in low-risk PTMC patients. The nomogram established in this study can help to determine the presence of adverse pathologic features in low-risk PTMC patients and assist clinicians in choosing a surgical or non-surgical treatment to avoid overtreatment and undertreatment.

## Data Availability

The datasets used and/or analysed during the current study available from the corresponding author on reasonable request.
